# lncRNA *CYTOR* Facilitates Osteogenic Differentiation of Human Periodontal Ligament Stem Cells by Modulating SOX11 via Sponging miR-6512-3p

**DOI:** 10.1155/2023/5671809

**Published:** 2023-03-03

**Authors:** Shaoqin Tu, Yihua Chen, Yi Feng, Zhili Kuang, Yuxuan Wang, Lin Chen, Zhihui Mai, Jiaming Wei, Sai Zhang, Yiting Shao, Hong Ai, Zheng Chen

**Affiliations:** ^1^Department of Stomatology, The Third Affiliated Hospital of Sun Yat-sen University, Guangzhou, China; ^2^Department of Stomatology, The Seventh Affiliated Hospital of Sun Yat-sen University, Shenzhen, China; ^3^Department of Stomatology, Huazhong University of Science and Technology Union Shenzhen Hospital (Nanshan Hospital), Shenzhen, China

## Abstract

Periodontal ligament stem cells (PDLSCs) are considered ideal cell sources for the regeneration of periodontal and alveolar bone tissue. Cytoskeleton Regulator RNA (*CYTOR*), a newly discovered long noncoding RNA, has been reported to function as competing endogenous RNA (ceRNA) and to be involved in many biological processes. However, its roles in PDLSC osteogenic differentiation remain unclear. Here, we firstly found *CYTOR* was mainly sublocalized in the cytoplasm of PDLSCs and *CYTOR* expression was increased during osteogenic differentiation of PDLSCs. By employing gain- and loss-of-function approaches, we then identified *CYTOR* overexpression promoted osteogenic differentiation of PDLSCs while *CYTOR* knockdown inhibited this process. Furthermore, bioinformatics analysis was utilized to show that both *CYTOR* and *SOX11* mRNA contained the same seed sites for miR-6512-3p, which was further confirmed by dual luciferase reporter assay and RNA-binding protein immunoprecipitation. Notably, *CYTOR* conferred its functions by directly binding to miR-6512-3p and an inverse correlation between *CYTOR* and miR-6512-3p on the level on SOX11 and osteogenic differentiation of PDLSCs was obtained. Additionally, miR-6512-3p could bind to *SOX11* mRNA 3′ UTR and repressed SOX11 expression. Moreover, level of SOX11 was significantly increased during osteogenic differentiation of PDLSCs. Knockdown of SOX11 attenuated the increasing effect of *CYTOR* overexpression on osteogenic differentiation of PDLSCs. Collectively, these data supported that *CYTOR* positively modulated the expression of SOX11 through competitively binding to miR-6512-3p, thus promoting osteogenic differentiation of PDLSCs. The *CYTOR*/miR-6512-3p/SOX11 axis could be a novel therapeutic target for periodontal regeneration medicine.

## 1. Introduction

Human periodontal ligament stem cells (PDLSCs) are a kind of mesenchymal stem cells which are derived from human periodontal ligament tissues. They were first isolated and cultured by Seo et al. [1]. Follow-up studies confirmed that PDLSCs display a high proliferative ability and have the potential to differentiate into several different cell types, including osteoblasts, cementoblasts, chondrocytes, and adipocytes [1, 2]. Periodontitis is a highly prevalent chronic inflammatory disease associated with loss of the periodontal tissues including the gingiva, periodontal ligament, cementum, and especially the alveolar bone around the teeth, which eventually lead to loss of teeth. PDLSCs are considered a promising cell population for alveolar bone regeneration therapy in periodontitis. In this respect, deeper understanding of the mechanism that governs the osteogenic differentiation of PDLSCs is urgently needed and would greatly facilitate the development of novel therapeutic approaches for tissue regeneration.

Long noncoding RNA (lncRNA) are a class of nonprotein coding transcripts larger than 200 nucleotides. Their importance in gene expression and key cellular processes have recently drawn wide attentions. Emerging evidences have found that lncRNAs are involved in numerous biological processes, such as transcription, posttranscription, and translational regulation of gene expression, thus governing development and metastasis of tumors, self-renewal, and differentiation of stem cells [3, 4]. Recently, efforts have also been made to clarify the roles of lncRNAs during osteogenic differentiation of PDLSCs. lncRNAs are differentially expressed during PDLSC osteogenic differentiation, indicating that lncRNAs may functionally be involved in this process [5, 6]. For example, long noncoding RNA taurine upregulated gene 1 (TUG1) promotes PDLSC osteogenic differentiation via binding Lin28A [7]. Huang et al. reported lncRNA Fer-1-like family member 4 (FER1L4) positively governs the osteogenic differentiation of PDLSCs through miR-874-3p and vascular endothelial growth factor A (VEGFA) [8]. Another lncRNA, antidifferentiation noncoding RNA (ANCR), which was downregulated during the process of stem cell differentiation, was found to suppress bone formation of PDLSCs via sponging miRNA-758 [9]. So far, although a few lncRNAs were reported to be positively or negatively involved in PDLSC osteogenic differentiation, the function of the vast majority of lncRNAs is largely unknown.

Cytoskeleton Regulator RNA (*CYTOR*), a novel intergenic lncRNA with 852 bp nucleotides, is located at chromosome 2p11.2 (87455455-87521518) and can be found in tissues, cells, serum, and exosomes. Emerging evidence suggests that *CYTOR* is closely involved in physiological processes such as cell proliferation and differentiation [10, 11]. It has been also reported that *CYTOR* takes part in the development of several cancers, such as colorectal cancer, lung adenocarcinoma, and glioma [12–14]. Yue et al. demonstrated that *CYTOR* was significantly upregulated in colon cancer and functioned as a competing endogenous RNA (ceRNA) to confer resistance to oxaliplatin-induced apoptosis [12]. A recent study found that *CYTOR* was highly expressed in RNA sequencing dataset of PDLSCs exposed to tensile loading and might be involved in orthodontic tooth movement [15]. However, the molecular functions and mechanisms of *CYTOR* in the osteogenic differentiation of PDLSCs remain unclear.

The present work was to explore whether lncRNA *CYTOR* was mechanistically involved in the osteogenic differentiation of PDLSCs. Our data proved that *CYTOR* was a positive regulator of PDLSC osteogenic differentiation and uncovered that *CYTOR* functioned as ceRNA to modulate SOX11 via sponging miR-6512-3p. Our findings offered novel ideas for the mechanism that underlies PDLSC osteogenic differentiation and could serve as novel therapeutic approaches for periodontal tissue regeneration.

## 2. Materials and Methods

### 2.1. Bioinformatics Analysis

To predict miRNAs which potentially interacted with *CYTOR*, LncBase v2.0 (http://www.microrna.gr/LncBase), StarBase v3.0 (http://starbase.sysu.edu.cn), and RNAhybrid (https://bibiserv.cebitec.uni-bielefeld.de/rnahybrid) were run, and a Venn diagram was used to intersect these potential miRNAs. For target genes of miR-6512-3p, TargetScan (http://www.targetscan. Org/vert_72), miRDB (http://mirdb.org/index.html), and StarBase v3.0 (http://starbase.sysu.edu.cn) were applied to predict the potential target genes of miR-6512-3p, respectively. The positively osteogenesis/odontogenesis regulation genes from Gene Ontology Term (GO: 0001649) were obtained. All these genes obtained above were intersected with a Venn diagram to find miR-6512-3p target genes related to osteogenic differentiation.

### 2.2. Cell Isolation and Culture

The healthy premolar or wisdom teeth were collected from healthy patients ranging from 18 to 25 years old for orthodontic purposes in the stomatology department of the Third Affiliated Hospital of Sun Yat-sen University. This study was approved by the ethics committee of the Third Affiliated Hospital of Sun Yat-sen University. All participants were informed the experimental principle and signed the informed consent at the beginning of the study. Human periodontal ligament stem cells were isolated and cultured as we previously reported [16]. In brief, the newly extracted teeth were transferred in *α*-MEM (Gibco, USA) with 5% penicillin/streptomycinn and the periodontal ligament tissues were obtained by scraping the middle third of teeth root surface after washing 6-8 times with phosphate-buffered saline (PBS; containing penicillin/streptomycin). Then, the tissue fragments were digested in 3 mg/mL collagenase type I (Roche Diagnostics Corp, USA) for 30 min at 37°C with 5% CO_2_. Afterwards, five times volume of medium was added to terminate the digestion. The solution was centrifuged at 1000 rpm/min for 5 min, and the spined cells and tissues were collected and cultured in primary growth medium consisting of *α*-MEM (Gibco, USA) supplemented with 20% fetal bovine serum (FBS; Gibco, USA) at 37°C in a humidified atmosphere of 5% CO_2_. Culture medium was changed every three days. Primary cells were passaged until they reached 80-90% confluency and then cultured in standard growth medium consisting of *α*-MEM (Gibco, USA) supplemented with 10% FBS (Gibco, USA) at 37°C in a humidified atmosphere of 5% CO_2_. The medium was changed every 2 days. Cells at passages 3-5 were used in subsequent experiments.

For osteogenic induction, the PDLSCs were cultured with osteogenic medium (OM) consisting of growth medium, 100 nmol/L dexamethasone (Sigma, USA), 50 *μ*g/mL ascorbic acid (Sigma, USA), and 10 mmol/L *β*-glycerophosphate (Sigma, USA) when cells reached 60-70% confluence. The medium was changed every 2 days.

### 2.3. Cell Transfection

Overexpression and knockdown *CYTOR* lentiviruses were designed and constructed by GeneChem (Shanghai, China) and were transfected into PDLSCs with HitransG P virus infection reagent (GeneChem, China) following the manufacturer's protocol. Briefly, *CYTOR* genomic DNA was inserted into (polyA-MCS-UBI) RV-SV40-EGFP-IRES-puromycin vector to construct *CYTOR* overexpression plasmid (over*CYTOR*), while three short hair RNAs (shRNAs) targeting *CYTOR* were, respectively, inserted into hU6-MCS-ubiquitin-EGFP-IRES-puromycin empty vectors to construct *CYTOR* knockdown plasmids (sh*CYTOR*). Empty overexpression vector was used as an overexpression negative control (overNC), and scramble shRNA were inserted into knockdown vector to construct knockdown negative control (shNC). All these plasmids were transfected into 293T cells to package their corresponding lentiviruses. For *CYTOR* overexpression or knockdown experiments, PDLSCs were transfected with over*CYTOR*, or sh*CYTOR*, or their control adenovirus as indicated, respectively. qRT-PCR and detection of GFP signal under inverted fluorescence microscope were applied to assess the transfection efficiency.

For miRNA modulation, PDLSCs cultured in odontoblastic induction medium or standard growth medium were transfected with miRNA mimic, inhibitor for miR-6512-3p, and their respective negative control (RiboBio, Guangzhou, China) as indicated, respectively. The transfection efficiency was validated by qRT-PCR.

For the effect of *CYTOR* on miR-6512-3p level, *CYTOR* mutant (only mutated the putative miR-6512-3p recognition element) overexpression was transiently transfected into PDLSCs. For *SOX11* knockdown, three small interfering RNA (siRNA) targeting *SOX11* (si*SOX11*) and their scramble siRNA control (siNC) were designed and generated by Gencefe (Jiangsu, China) and transfected using Liposomal Transfection Reagent (Yeasen, China) following the manufacturer's protocol. The sequences used were presented in Table 1. The silencing efficiency and specificity of all siRNAs were validated by qRT-PCR.

### 2.4. Fluorescent In Situ Hybridization (FISH)

Cy3-labeled probes targeting *CYTOR* and U6 were designed and generated by RiboBio Company (RiboBio, China). U6 probes were used as internal references. FISH was performed with a Fluorescence in Situ Hybridization Kit (RiboBio, China) as per the manufacturer's instructions. In brief, PDLSCs were cultured on cover glass and fixed by 4% paraformaldehyde for 20 mins at room temperature. Fixed cells were then rinsed with PBS and permeabilized by a precold non-ionic detergent (0.5% Triton X-100) in PBS for 15 min on ice. Afterwards, cells were proceeded to blocking stage by incubating in prehybridization buffer with blocking solution for 30 mins at 37°C and then incubated in prewarmed hybridization buffer with 0.5 *μ*M FISH probe mix overnight at 37°C in the dark. Cells were then rinsed with 4x SSC at 42°C to reduce the background. Nuclei were counterstained with 4′,6-diamidino-2-phenylindole (DAPI) for 10 mins. Stained samples were mounted with antifade mounting medium. Images were captured by using a confocal laser scanning microscope (Leica, Germany).

### 2.5. Quantitative Real-Time Polymerase Chain Reaction (qRT-PCR)

qRT-PCR was run as we previously reported [17]. Briefly, total RNA representing the indicated time points were extracted with NucleoZOL reagent (MACHEREY-NAGEL, Germany) and were separated in small RNA (10-200 nt) and large RNA (>200 nt) in two fractions if needed following the manufacture's protocol. The quality and concentration of RNA were further measured by Thermo NanoDrop 2000 spectrophotometer (Thermo Scientific, USA). Then, 1 *μ*g total RNA was reversely transcribed into cDNA using PrimeScriptTM RT Master Mix (TaKaRa, Japan) on Thermal Cycler (Thermo Scientific, USA). PCR amplification were run with qPCR SYBR Master Mix (Yeasen, China) on the LightCycler 96 Real-time fluorescence quantitative PCR Detection System (Roche, Switzerland). Specific primers for lncRNA and mRNA were synthesized by Synbio Technologies (Suzhou, China) and listed in Supplementary Table 1. The amplification reactions were run with 40 thermocycles of 60 s at 94°C, 30 s at 55°C, and 30 s at 72°C.

For miR-6512-3p analysis, miRNA was treated with DNase 1 to eliminate genomic DNA. Then, cDNAs were synthesized by a specific RT primer (RiboBio, Guangzhou, China) and qRT-PCR was carried out with Bulge-LoopTM miRNA qRT-PCR starter kit (RiboBio, Guangzhou, China). *β*-Actin was used as endogenous normalization control for lncRNAs and mRNAs, while U6 was used for miRNA. The data were analyzed using the 2^−*ΔΔ*Ct^ relative expression method as described previously, and were displayed as fold change compared to respective control conditions. All experiments were repeated three times.

### 2.6. Western Blot Analysis

Western blot was performed as described before [18]. Total proteins were extracted from the cells by RIPA lysis buffer (Beyotime, China) with 1% protease inhibitor (CoWin Biosciences, China). The protein concentrations were then calculated by using the BCA kit (Beyotime, China) according to the manufacturer's recommendations. Protein denaturation was performed at 100°C for 10 min with the protein concentration at 2 *μ*g/*μ*L. Then, 25 *μ*g of total protein was electrophoresed by 10% sodium dodecyl sulfate polyacrylamide gel electrophoresis (SDS-PAGE) and transferred onto polyvinylidene fluoride (PVDF) membranes (Merck Millipore, Ireland). The membranes were blocked by 1X TBST with 5% skim milk powder (BD, USA) for 2 hours at room temperature and then incubated in diluted primary antibodies overnight at 4°C with gentle rotation. Primary antibodies are listed as follows: rabbit SOX11 polyclonal antibody (Proteintech, cat#29395-1-AP, 1 : 1000), rabbit osteocalcin antibody (Affinity, cat#DF12303, 1 : 1000), RUNX2 (D1L7F) Rabbit mAb (Cell Signaling Technology, cat#12556S, 1 : 1000), rabbit Osterix antibody (Affinity, cat#DF7731, 1 : 1 000), and rabbit beta Actin Antibody (Affinity, cat#AF7018, 1 : 3000). The washed membranes were then immunoblotted with HRP-conjugated goat anti-rabbit immunoglobulin-G (IgG) secondary antibody (Beyotime, cat#A0208, 1 : 2000) for 1 hour at room temperature. Immunodetection was carried out with the enhanced chemiluminescence (ECL) reagents (Affinity, cat#KF005, Liyang, China) after washed by TBST for 3 times. ImageJ software was applied for the gray intensity analysis. All experiments were repeated three times.

### 2.7. Alizarin Red Staining

Alizarin red staining was applied to assess the mineral nodule formation ability as described previously [17]. The culture PDLSCs were fixed in 4% paraformaldehyde for 20 minutes at room temperature and were then stained by Alizarin Red S Solution (Cyagen, cat#ALIR-10001, Guangzhou, China) for 5 minutes at room temperature. Excessive dye was removed by several washes in deionized water. Then, the staining was imaged with an inverted light microscope and proceed to semiquantification. To quantify the matrix mineralization, the stain was eluted by 100 mM cetylpyridinium chloride for 10 minutes with gentle rotation and spectrophotometric absorbance at 562 nm was detected. ARS intensity relative to the control group was calculated after normalization to the total protein content.

### 2.8. Alkaline Phosphatase (ALP) Activity and Staining Assay

The PDLSCs of indicated groups were cultured with OM for 7 days in 24-well plates at a density of around 5 × 10^4^ cells/well. Then, the ALP activity and staining were assessed by Alkaline Phosphatase Assay Kit (Beyotime, cat#P0321S, Shanghai, China) and BCIP/NBT Alkaline Phosphatase Color Development Kit (Beyotime, cat#C3206, Shanghai, China) according to the manufacturer's protocols. For ALP activity, cells were lysed with inhibitor-free Western and IP lysis buffer (Beyotime, China). The supernatant was incubated with 10 *μ*L *p*-nitrophenol solution for 10 min at 37°C, and the spectrophotometric absorbance at 405 nm was recorded after terminating reaction. The total protein amount detected with the BCA assay as described above was used to normalize the ALP activity. For ALP staining, cells were rinsed with PBS and fixed with 4% paraformaldehyde for 20 min at room temperature. After washing with PBS for 3 times, the BCIP/NBT ALP staining working solution was added and incubated for 30 mins at room temperature. Afterwards, cells were rinsed by purified water and observed under a light microscope.

### 2.9. Dual Luciferase Reporter Gene Assay

Both the wild-type (wt) and mutant type (mut) sequences of the predicted miR-6512-3p binding sites in *CYTOR* and *SOX11* mRNA 3′ UTR were designed and cloned downstream of the luciferase gene in pmirGLO luciferase vectors (Yeasen, China). The plasmids and miR-6512-3p mimic or mimic control were cotransfected into 293T cells using the Liposomal Transfection Reagent (Yeasen, China). Firefly and Renilla luciferase activities were then measured consecutively by Dual Luciferase Reporter Gene Assay Kit (Yeasen, China) following the manufacturer's recommendations 48 hours after transfection. All experiments were repeated three times.

### 2.10. RNA-Binding Protein Immunoprecipitation (RIP) Assay

RIP assay was performed with the Magna RIP™ RNA-Binding Protein Immunoprecipitation Kit (Millipore, USA) according to the manufacturer's protocol with the anti-Argonaute-2 (AGO2) antibody and negative control antibody Normal Mouse IgG. Anti-SNRNP70 and negative control Normal Rabbit IgG served as controls for the RIP assay. Cells were collected and lysed by RIP lysis buffer with proteinase and RNase inhibitor when reaching 80-90% confluence. The cell lysate was then incubated with magnetic beads precoated with relevant antibody. qRT-PCR was applied as described above to detect the enrichment of *CYTOR* and miR-6512-3p with U1 as endogenous normalization control. All experiments were repeated three times.

### 2.11. Statistical Analysis

All the statistical analyses were performed with GraphPad Prism 8.0 software. Data was presented as mean value ± standard error of the mean (mean ± SEM) from 3 independent biological repeats. The comparisons between two groups were analyzed by unpaired, two-tailed Student's *t* test. One-way ANOVA test was applied to test for statistical significance among more sample groups. *p* value < 0.05 was considered statistically significant.

## 3. Results

### 3.1. Long Noncoding RNA *CYTOR* Is Mainly Expressed in the Cytoplasm of PDLSCs and Facilitates the Osteogenic Differentiation of PDLSCs

**Figure 1:**
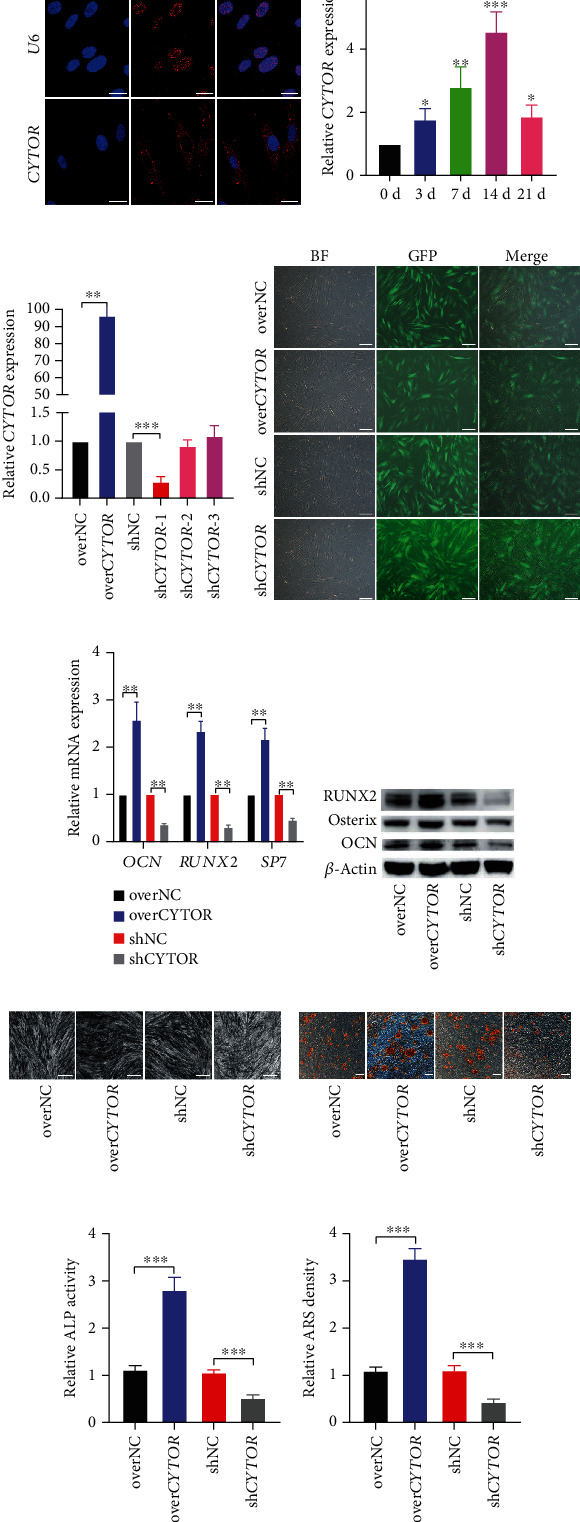
Long noncoding RNA *CYTOR* is mainly expressed in the cytoplasm of PDLSCs and facilitates the osteogenic differentiation of PDLSCs. (a) The expression and subcellular location of *CYTOR* in PDLSCs was observed by RNA fluorescence in situ hybridization. U6 was a reference gene which mainly expressed in the nuclei. Probe against U6 as a positive control. Cell nuclei were counterstained with DAPI (blue). Scale bar, 20 *μ*m. (b) Relative level of *CYTOR* at indicated timepoints during osteogenic differentiation of PDLSCs were detected by qRT-PCR. (c) RNA level of *CYTOR* of PDLSCs treated with lentiviruses as indicated was measured by qRT-PCR. (d) The GFP signals were detected by inverted fluorescence microscope. (e) The mRNAs levels of osteogenic-related markers were determined by qRT-PCR. (f) The protein levels of osteogenic-related markers were measured by western blot. (g, i) The ALP activity was determined by ALP staining (g) and ALP measurement (i). (h) The mineralized nodules were assessed by Alizarin red staining. (j) Semiquantification of mineralized nodules. ^∗^*p* < 0.05, ^∗∗^*p* < 0.01, and ^∗∗∗^*p* < 0.001, compared with the control group as indicated.

To clarify the characteristic of *CYTOR* in PDLSCs, we firstly applied RNA fluorescence in situ hybridization to detect the subcellular localization of *CYTOR*. We found *CYTOR* was mainly sublocalized in the cytoplasm of PDLSCs, compared to U6 which was a reference gene and only expressed in the nucleus (Figure 1(a)). Then, qRT-PCR was carried out to evaluate the expression pattern of *CYTOR* during osteogenic differentiation of PDLSCs. The results showed that the *CYTOR* level presented an osteogenic induction time-dependent increasing trend (Figure 1(b)).

Given that *CYTOR* is highly expressed during osteogenic differentiation, we performed in vitro *CYTOR* gain-of-function and loss-of-function approaches in PDLSCs to clarify whether *CYTOR* could promote osteogenic differentiation of PDLSCs. The full long sequences of *CYTOR* and three short hairpin RNAs (shRNAs) targeting the sequences of *CYTOR* were used to construct *CYTOR* overexpressed (over*CYTOR*) and knockdown (sh*CYTOR*) plasmid, respectively. The overexpressed empty vector (overNC) and the knockdown vector (shNC) containing the scramble sequences served as the negative control. These plasmids were packaged into lentiviruses to infect PDLSCs. qRT-PCR was applied to test the transfection efficiency. Results indicated that over*CYTOR* could effectively overexpressed *CYTOR* in PDLSCs and the most efficient sh*CYTOR* was chosen for the following experiments (Figure 1(c)). Also, the GFP signals in PDLSCs after transfection were detected by inverted fluorescence microscope to further verify the transfection efficiency (Figure 1(d)). Thus, we successfully constructed over*CYTOR* and sh*CYTOR* lentivirus to manipulate *CYTOR* level for further investigation.

We transfected PDLSCs with over*CYTOR* or sh*CYTOR* lentivirus or their respective negative control and cultured them in an osteogenic medium. ALP activity/staining, Alizarin Red staining, qRT-PCR, and western blot were performed to assess the osteogenic ability of PDLSCs. Results of qRT-PCR and western blot showed that *CYTOR* overexpression significantly upregulated the mRNA and protein levels of osteogenic markers (OCN, Osterix, and RUNX2), while knockdown of *CYTOR* apparently downregulated these osteogenic-related genes (Figures 1(e) and 1(f)). ALP activity was remarkably enhanced in over*CYTOR* transfected group but was noticeably reduced in the sh*CYTOR* group (Figures 1(g) and 1(i)). Meanwhile, more mineral nodules were detected in the over*CYTOR* group while *CYTOR* knockdown inhibited the formation of mineral nodules (Figures 1(h) and 1(j)). These results suggest that *CYTOR* facilitates osteogenic differentiation of PDLSCs.

### 3.2. *CYTOR* Directly Binds to miR-6512-3p

**Figure 2:**
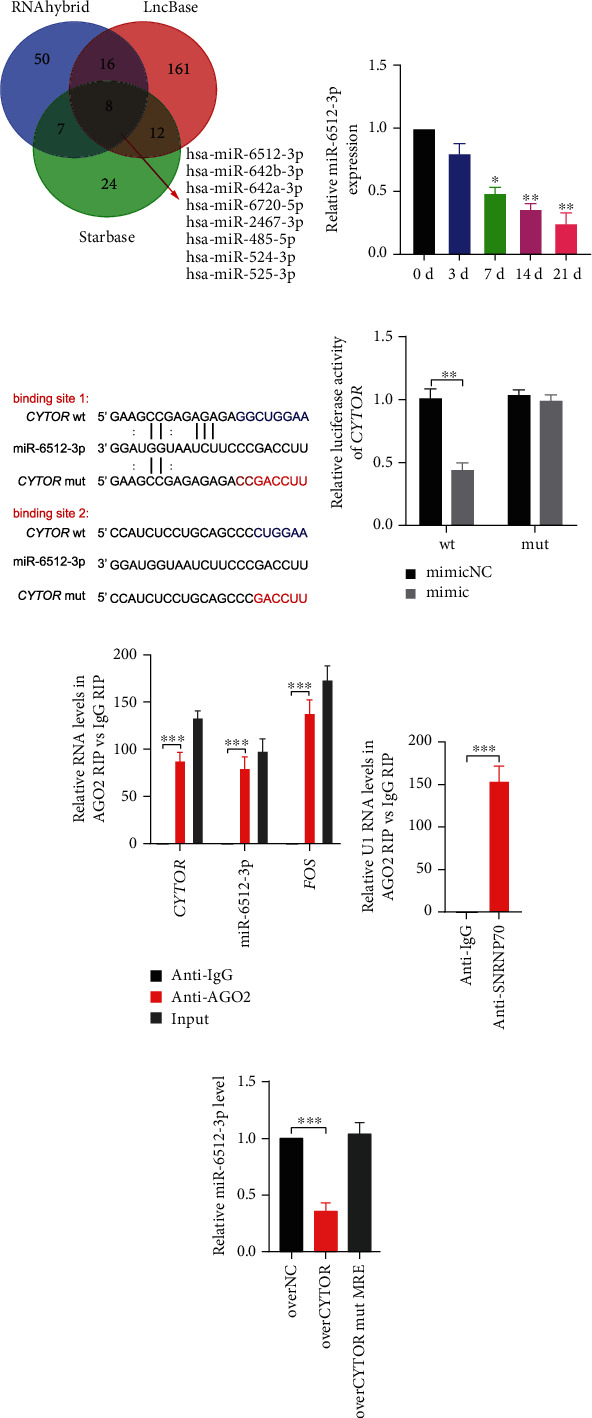
*CYTOR* directly binds to miR-6512-3p. (a) The Venn diagram showed the overlap of miRNAs which had potential binding sequences of *CYTOR*, based on several online systems. (b) The miR-6512-3p levels throughout the osteogenic differentiation time course were detected by qRT-PCR. (c) The putative binding sites (blue color) and mutated binding sites (red color) between *CYTOR* and miR-6512-3p were shown. The dual luciferase reporter constructs containing the wild type (*CYTOR*-wt) or mutant *CYTOR* (*CYTOR* mut). (d) *CYTOR*-wt or *CYTOR* mut was cotransfected into 293T cells with miR-6512-3p mimic or negative control, and Luciferase activity was detected. (e) RNA-binding protein immunoprecipitation (RIP) assay was performed by using the AGO2 antibody in PDLSCs. The enrichment of *CYTOR* and miR-6512-3p in AGO2-immunoprecipitated complexes were assessed by following qRT-PCR. (f) Positive control of RIP procedure. Anti-SNRNP70 served as a positive control antibody. (g) Level of miR-6512-3p was assessed by qRT-PCR in over*CYTOR* or over*CYTOR* mut MRE-treated hPDLSCs. ^∗^*p* < 0.05, ^∗∗^*p* < 0.01, and ^∗∗∗^*p* < 0.001, compared with the control group as indicated.

Previous studies demonstrated that certain cytoplasmic lncRNAs could serve as competitive endogenous RNAs (ceRNAs) to sponge miRNA by competing for miRNA binding, thereby derepressing miRNA targets. It was an important mechanism that how lncRNAs in the cytoplasm functioned [4, 19, 20]. As aforementioned FISH result, *CYTOR* was mainly expressed in the cytoplasm of PDLSCs. Thus, we wonder whether *CYTOR* promoted osteogenic differentiation of PDLSCs via interacting with miRNAs. To test this hypothesis, we first applied several online systems, including LncBase, StarBase, and RNAhybrid, to predict miRNAs which have potential binding sequences of *CYTOR*. A Venn diagram was used to intersect these potential miRNAs, and we got 8 overlapped miRNAs, including hsa-miR-6512-3p, hsa-miR-642b-3p, hsa-miR-642a-3p, hsa-miR-525-3p, hsa-miR-2467-3p, hsa-miR-485-5p, hsa-miR-524-3p, and hsa-miR-6720-5p (Figure 2(a)). Results of our preliminary experiment showed that among these miRNAs, only the expression of hsa-miR-6512-3p was reduced and could be inhibited by overexpressed *CYTOR* during PDLSC osteogenic differentiation (supplement figure 1A-B). Thus, hsa-miR-6512-3p were chosen. Then, we detected miR-6512-3p level during osteogenic differentiation of PDLSCs via qRT-PCR. Results indicated that expression of miR-6512-3p reduced during osteogenic process, presenting an opposite character to *CYTOR* level (Figure 2(b)). Further, dual luciferase reporter gene assay was carried out to evaluate the binding sites between *CYTOR* and miR-6512-3p. We constructed *CYTOR* wild type (*CYTOR* wt) luciferase reporter plasmid containing miR-6512-3p potential binding sites and *CYTOR* mutant type (*CYTOR* mut) where the putative binding sequences were mutated as Figure 2(c) shown. We found that miR-6512-3p mimic apparently reduced the luciferase activity of *CYTOR* wt compared to its negative control. However, no significant difference was observed in *CYTOR* mut (Figure 2(d)). Interestingly, we further found that *CYTOR* inhibited the level of miR-6512-3p through the binding sites (Figure 2(g)). Moreover, it is well known that Argonaute 2 (AGO2) is a main component of microRNA-related silencing complex which generally contains miRNAs and their interacting RNA component [21]. Thus, RNA-binding protein immunoprecipitation (RIP) assay was performed by using the AGO2 antibody in PDLSC lysates and the enrichment of *CYTOR* and miR-6512-3p in AGO2-immunoprecipitated complexes were further assessed by qRT-PCR. We found that both *CYTOR* and miR-6512-3p were preferentially enriched in anti-AGO2 immunoprecipitates compared to anti-IgG immunoprecipitates. Using RIP primers against human FOS, we verified the successful immunoprecipitation of AGO2-associated RNA (Figure 2(e)). Besides, anti-ANRNP70 was used as a positive control for the RIP procedure and U1 snRNA was enriched at a greater level than that of anti-IgG (Figure 2(f)). Taken together, these data support that *CYTOR* could directly interact with miR-6512-3p in the cytoplasm of PDLSCs.

### 3.3. *CYTOR* Facilitates Osteogenic Differentiation of PDLSCs via Competitively Binding to miR-6512-3p

**Figure 3:**
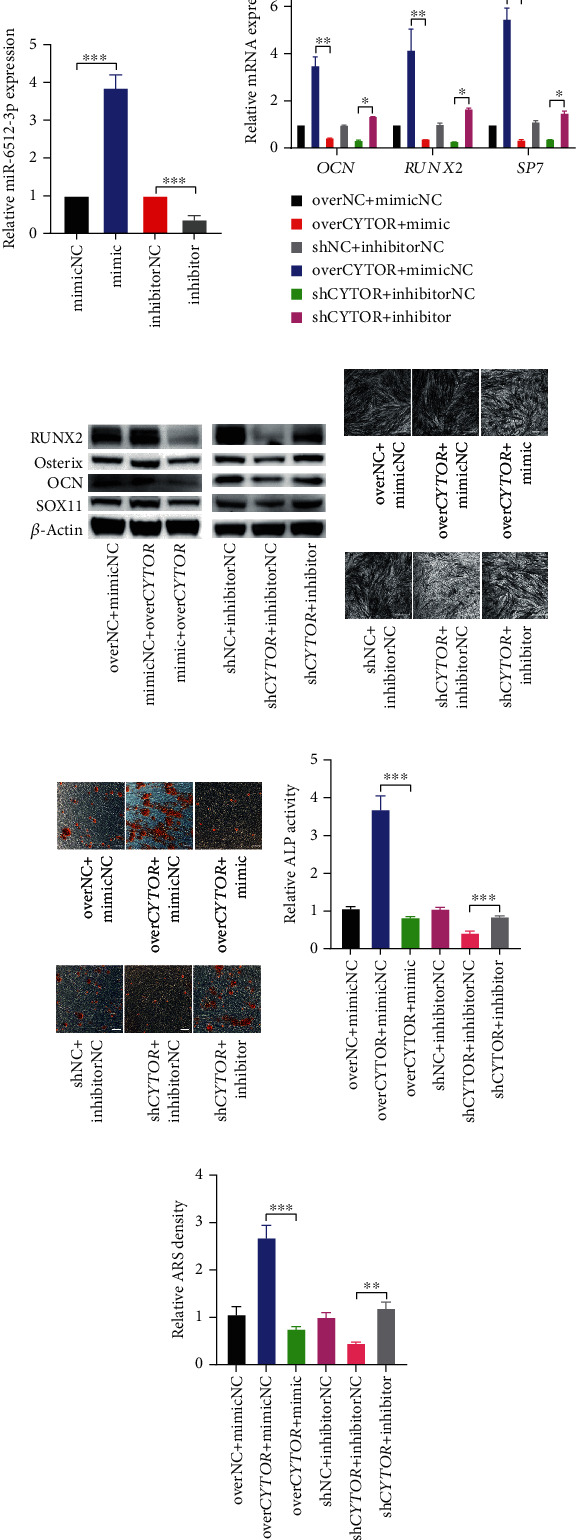
*CYTOR* facilitates osteogenic differentiation of PDLSCs via competitively binding to miR-6512-3p. (a) The miR-6512-3p expression was determined by qRT-PCR after transfection of miR-6512-3p mimic and miR-6512-3p inhibitor, compared with their respective control group. (b, c) The mRNA (b) and protein (c) levels of osteogenic related marker genes (*RUXN2*, Osterix, and *OCN*) were detected by western blot in indicated groups. (d, f) ALP staining (d) and ALP activity (f) in PDLSCs of indicated groups and cultured with OM for 7 days. (e) The formation of mineralized nodules was detected by Alizarin Red staining. (g) Semiquantification of (e). ^∗^*p* < 0.05, ^∗∗^*p* < 0.01, and ^∗∗∗^*p* < 0.001, compared with the group as indicated.

To further assess whether the binding between *CYTOR* and miR-6512-3p influence osteogenic differentiation of PDLSCs, we firstly applied miR-6512-3p mimic and miR-6512-3p inhibitor to manipulate the expression of miR-6512-3p in PDLSCs. Results of qRT-PCR showed that miR-6512-3p mimic/inhibitor could effectively manipulate the level of miR-6512-3p (Figure 3(a)). Then, we cotransfected miR-6512-3p mimic or inhibitor with over*CYTOR* or sh*CYTOR* or their corresponding control to upregulate or downregulate *CYTOR* and miR-6512-3p in the same time. We performed qRT-PCR and western blot to examine the mRNAs and proteins levels of osteogenic-related markers. Results indicated that miR-6512-3p overexpression could attenuate the up-regulation effect of *CYTOR* overexpression on osteogenic-related genes, while inhibition of miR-6512-3p could rescue the repression effect of *CYTOR* knockdown on osteogenic related genes (Figures 3(b) and 3(c)). ALP staining, ALP activity, and Alizarin Red staining also showed similar results (Figures 3(d)–3(g)). These data suggest that *CYTOR* facilitates osteogenic differentiation of PDLSCs via competitively binding to miR-6512-3p.

### 3.4. *CYTOR* Relieves Repression of miR-6512-3p on SOX11

**Figure 4:**
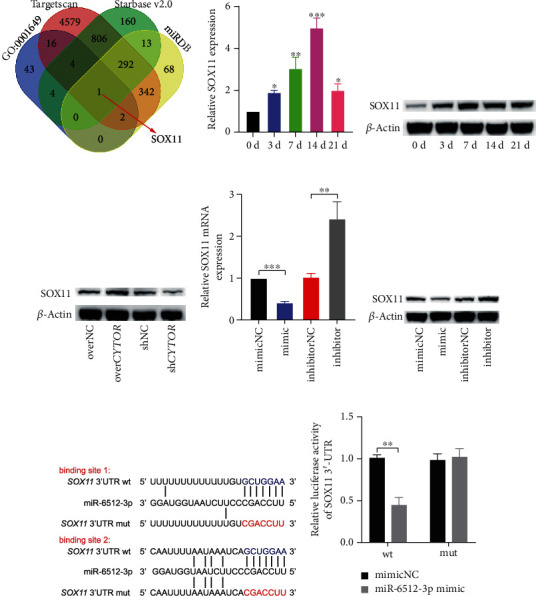
SOX11 is upregulated during osteogenic differentiation of PDLSCs and is modulated by *CYTOR* and miR-6512-3p. (a) A Venn diagram of the intersections between the target genes of miR-6512-3p predicted on several online systems (TargetScan, StarBase, and miRDB) and the highly expressed genes from GO:0001649. (b, c). *SOX11* mRNA (b) and protein (c) levels were determined by qRT-PCR and western blot during osteogenic differentiation of PDLSCs. (d) Effects of overexpression or knockdown of *CYTOR* on the protein level of SOX11. (e, f) The mRNA and protein levels of *SOX11* were evaluated by qRT-PCR and western blot in PDLSCs after transfection of miR-6512-3p mimic/inhibitor or their respective control. (g) Diagrammatic sketch of the binding sequences for the wt (blue) and mut (red) of *SOX11* mRNA 3′ UTR associated with miR-6512-3p. (h) Luciferase activity of *SOX11* mRNA 3′ UTR wt/mut cotransfected with miR-6512-3p mimic or mimic NC. ^∗^*p* < 0.05, ^∗∗^*p* < 0.01, and ^∗∗∗^*p* < 0.001. All tests were performed at least three times, and the values are presented as the mean ± SEM.

Bioinformatics analysis including TargetScan, StarBase, and miRDB were run to predict potential targets of miR-6512-3p. These intersect genes were further compared with the genes involved in the positive regulation of osteoblast differentiation from the Gene Ontology term (GO: 0001649), after which, one intersection *SOX11* was found (Figure 4(a)). Therefore, we pursued *SOX11* as a primary candidate for further investigation.

First, we applied qRT-PCR and western blot to assess the change of SOX11 through the osteogenic induction course of PDLSCs. We observed that both the mRNA and protein level of *SOX11* were highly expressed during osteogenic process (Figures 4(b) and 4(c)). Next, we evaluated the potential repressive effect of miR-6512-3p on *SOX11* in PDLSCs. As expected, miR-6512-3p mimic inhibited the expression of *SOX11* in PDLSCs in terms of mRNA and protein levels, while miR-6512-3p inhibitor increased *SOX11* level at both mRNA and protein level (Figures 4(e) and 4(f)). Furthermore, we constructed dual luciferase reporters containing wild-type and mutated binding sites in 3′ UTR region of *SOX11* mRNA to explore the binding between miR-6512-3p and *SOX11* mRNA 3′ UTR. Results of dual luciferase reporter gene assay showed that compared to negative control, transfection of miR-6512-3p mimic remarkably reduced the luciferase activity of reporters containing *SOX11* wt, instead of *SOX11* mut (Figures 4(g) and 4(h)). Moreover, effects of overexpression or knockdown of *CYTOR* on the protein level of SOX11 during osteogenic differentiation of PDLSCs were detected. We found *CYTOR* overexpression upregulated the level of SOX11, while knockdown of *CYTOR* inhibited the expression of SOX11 (Figure 4(d)). Last but not least, we found overexpression of miR-6512-3p weakened the upregulation effect of over*CYTOR* on SOX11 level, and inhibition of miR-6512-3p sequestered the inhibition effect of *CYTOR* knockdown on SOX11 expression (Figure 3(c)). Thus, reciprocal effect on SOX11 level existed between *CYTOR* and miR-6512-3p. Collectively, these data suggest that *CYTOR* relieves repression of miR-6512-3p on SOX11.

### 3.5. *CYTOR* Promotes Osteogenic Differentiation of PDLSCs through Regulating SOX11

**Figure 5:**
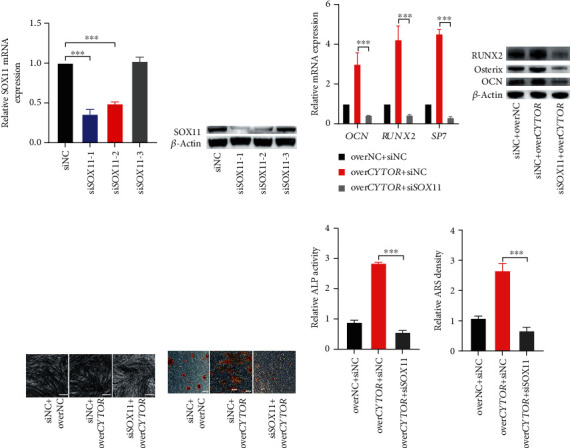
*CYTOR* promotes osteogenic differentiation of PDLSCs through regulating SOX11. (a, b) Verification of the effect of knockdown on *SOX11*. The mRNA and protein levels of *SOX11* was measured by qRT-PCR and western blot after silencing *SOX11* by si*SOX11*. We used si*SOX11*-1 to silence *SOX11* in the subsequent experiments. (c, d) qRT-PCR and western blot were conducted to detected the mRNA (c) and protein (d) levels of osteogenic related markers in PDLSCs after cotransfected with si*SOX11* and over*CYTOR* or their negative controls followed by 14 days' osteogenic differentiation. (e) ALP staining was performed. (g) ALP activity of indicated groups were detected after 7 days' osteogenic induction. (f) Alizarin Red staining was performed to detect mineralized nodules formation of indicated groups. (h) Semiquantification of (f). ^∗∗∗^*p* < 0.001, compared with the group as indicated.

Based on these above results, we speculated that *CYTOR* might regulate SOX11 expression through sponging miR-6512-3p to facilitate osteogenic differentiation of PDLSCs. To further confirm the function of SOX11 in osteogenic differentiation, RNA interference (RNAi) was applied. Three human *SOX11*-specific small interference RNA (si*SOX11*) or scramble small RNA (siNC) were transfected into PDLSCs. qRT-PCR and western blot were performed to verify the knockdown efficiency of *SOX11*. As expected, both the mRNA and protein levels of *SOX11* were efficiently silenced by si*SOX11* and we used si*SOX11*-1 to knock down *SOX11* in further investigation (Figures 5(a) and 5(b)). We cotransfected over*CYTOR* with si*SOX11* or their negative controls into PDLSCs and evaluated the osteogenic capability. Results of western blot showed that silencing *SOX11* attenuated the increasing effect of *CYTOR* overexpression on protein levels of osteogenic related markers in PDLSCs (Figures 5(c) and 5(d)). The ALP activity presented a similar result (Figures 5(e) and 5(g)). Besides, Alizarin Red staining showed that effect of overexpression *CYTOR* on mineralized nodule formation could be weakened by silencing *SOX11* (Figures 5(f) and 5(h)). Taken together, our results suggest that *CYTOR* enhances osteogenic differentiation of PDLSCs via modulating SOX11.

## 4. Discussion

**Figure 6:**
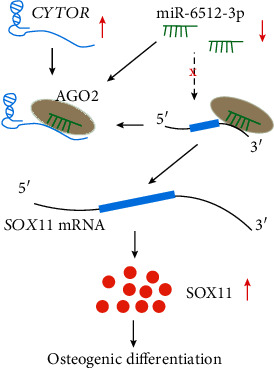
Schematic diagram of potential mechanisms involved in *CYTOR*-mediated osteogenic differentiation of PDLSCs. *CYTOR* acted as a molecular sponge to restrain the bioactivity of miR-6512-3p, resulting in increased miR-6512-3p targeting SOX11 expression, leading to the enhancement of osteogenic differentiation of PDLSCs.

Understanding the fine mechanisms modulating the osteogenic differentiation of PDLSCs is of paramount importance to develop regenerative therapies. This study revealed that long noncoding RNA *CYTOR* promoted PDLSC osteogenic differentiation by upregulating SOX11 via sponging miR-6512-3p (Figure 6).

Recently, emerging evidence have revealed that lncRNAs are of paramount importance in regulation of stem cell differentiation, especially in osteogenic differentiation. Osteogenic differentiation-associated lncRNAs and investigation of their biological and molecular functions are crucial to offer new ideas for the development of novel strategy for regeneration medicine. Additionally, information regarding the expression dynamics of lncRNAs and their subcellular localization are critical to help clarify biological functions and molecular mechanisms. RNA fluorescence in situ hybridization (FISH) is a kind of RNA visualization method, and it is vastly applied to detect subcellular distribution of RNA, including lncRNA. In present study, we applied RNA FISH and found *CYTOR* was mainly distributed in the cytoplasm of PDLSCs. This is consistent with previous results reported in HeLa cells [10]. ALP activity, expression of RUNX2, Osterix, OCN, and calcified nodules detected by Alizarin red are generally regarded as osteogenic related differentiation markers [18]. They are vastly used to assess osteogenic capability. Here, we found that level of *CYTOR* was increased during PDLSC osteogenic differentiation, and subsequent gain- and loss-of-function experiments demonstrated that *CYTOR* played an important role in osteogenic differentiation of PDLSCs. In specific, *CYTOR* overexpression could promote PDLSC osteogenic differentiation while knockdown of *CYTOR* attenuated the osteogenic ability of PDLSCs. Thus, we found a new PDLSC-associated lncRNA in osteogenic differentiation.

Accumulating evidence have suggested that cytoplasmic lncRNAs may serve a function broadly as competing endogenous RNAs (ceRNAs). In this scenario, cytoplasmic lncRNAs sequester miRNAs not only to reduce their availability to Argonaute-2/RNA-induced silencing complex (AGO2/RISC) but also to inhibit the expression of miRNA, thus relieving numerous instances of miRNA-mediated translational repression. Such ceRNA mechanism has been implicated as one of the main working mechanisms of cytoplasmic lncRNAs [4, 17, 19–21]. Given that *CYTOR* mainly sublocalized in the cytoplasm of PDLSCs, we next focused on the ceRNA mechanisms. Bioinformatics analysis, dual luciferase activity gene assay, and RIP assay are generally applied to predict and test the binding among these RNA molecules [4, 17]. In this study, we found that both *CYTOR* and *SOX11* shared the same seed site for miR-6512-3p, and they could directly bind to miR-6512-3p. Moreover, our subsequent functional studies demonstrated that *CYTOR* conferred its functions by directly binding to miR-6512-3p, and an inverse correlation between *CYTOR* and miR-6512-3p on the level on SOX11 and osteogenic differentiation of PDLSCs was obtained. In addition, it is known that the inhibitory effect of lncRNA on miRNA level will disappear if the miRNA recognition elements in lncRNA are mutated. Thus, the lncRNA could also direct miRNA degradation in a sequencing-specific and binding-site-dependent manner [4, 14, 19]. In our study, overexpression CYTOR reduced the level of miR-6512-3p, and this reduction effect could be rescued by mutating the miR-6512-3p binding sites in the sequences of CYTOR. It is consistent with previous results as reported [14, 17]. We speculate that the binding between CYTOR and miR-6512-3p leads to the degradation of miR-6512-3p, and the detail mechanism requires further research. Therefore, the crosstalk among *CYTOR*, miR-6512-3p, and SOX11 were attained and could be well explained by ceRNA regulation. Recently, circular RNAs (circRNAs), another kind of noncoding RNA, have also been reported to act as ceRNA to regulate osteogenic differentiation of PDLSCs [22]. Therefore, the ceRNA networks governing PDLSC osteogenic differentiation are much more complicated than what we know. More intense efforts are required on this topic in the future research.

Furthermore, miRNAs are the most widely studied noncoding RNAs and can also exert proosteogenic or antiosteogenic effects. In present study, we revealed that miR-6512-3p was significantly decreased during osteogenic differentiation of PDLSCs, and miR-6512-3p attenuated PDLSCs osteogenic differentiation. What is more, miR-6512-3p could bind to *SOX11* mRNA 3′ UTR and repressed SOX11 expression at the posttranscriptional level, thus repressing osteogenic differentiation of PDLSCs. SOX11 has been reported to be involved in neural development and organogenesis during development [23]. The *Sox11* knockout mice are known to exhibit developmental defects of craniofacial and skeletal malformations [24]. It was also reported that administration of allogenic Sox11-modified mesenchymal stem cells (MSCs) could accelerate bone fracture healing [25], and Sox11 could mediate Wnt7b-induced bone formation enhancement via enhances both self-renewal and osteogenic differentiation of bone derived-mesenchymal stem cells (BMSCs) [26]. In the current work, we found that level of SOX11 was significantly increased during osteogenic differentiation of PDLSCs. Besides, silencing of SOX11 attenuated the increasing effect of *CYTOR* overexpression on osteogenic differentiation of PDLSCs. These findings are consistent with the notion that SOX11 has a positive influence on osteogenic differentiation and expand our knowledge of SOX11 in PDLSCs. Previous studies have revealed that SOX11 has a number of direct transcriptional targets (*SP7*, RUNX2) which contribute to osteoblast differentiation [25, 26]. Nevertheless, it is currently unclear how SOX11 functions in the program of PDLSCs osteogenic differentiation, and further experiments are required.

Collectively, our findings indicate that *CYTOR* performed a pivotal role in PDLSC osteogenic differentiation. Namely, we found that *CYTOR* promoted osteogenic differentiation of PDLSCs by functioning as a ceRNA to enhance SOX11 level by sponging miR-6512-3p (Figure 6). The *CYTOR*/miR-6512-3p/SOX11 axis might be a novel target for bone regeneration.

## Figures and Tables

**Table 1:** Primer sequences used in this study.

	Sense (5′-3′)
sh*CYTOR*-1	GGCTTGAACATTTGGTCTT
sh*CYTOR*-2	TCTACTCATGCCCAAAGTT
sh*CYTOR*-3	GCCTCCATCCACATTCCAA
si*SOX11*-1	CCGCCUCUACUACAGCUUCAATT
si*SOX11*-2	CGCCAGCCAGAGCCCAGAGAATT
si*SOX11*-3	AGACGGUCAAGUGCGUGUUUCTT

## Data Availability

Data will be made available on request.

## Supplementary Materials

Supplementary Materials Supplementary Figure 1: miR-6512-3p was downregulated in hPDLSCs after osteogenic induction and could be reduced by overexpression *CYTOR*. (A) Relative expression of eight potential miRNAs in hPDLSCs cultured in growth medium (GM) and osteogenic medium (OM) for 14 days were detected by qRT-PCR. (B) Levels of these eight miRNAs in hPDLSCs treated with indicated lentiviruses were detected by qRT-PCR. ^∗∗^*p* < 0.01, compared with the indicated control group. Supplementary Table 1: primers for quantitative real-time polymerase chain reaction.
