# Corrigendum to “lncRNA *CYTOR* Facilitates Osteogenic Differentiation of Human Periodontal Ligament Stem Cells by Modulating SOX11 via Sponging miR-6512-3p”

**DOI:** 10.1155/sci/9825165

**Published:** 2025-09-15

S. Tu, Y. Chen, Y. Feng, et al., “lncRNA *CYTOR* Facilitates Osteogenic Differentiation of Human Periodontal Ligament Stem Cells by Modulating SOX11 via Sponging miR-6512-3p,” *Stem Cells International* 2023 (2023): 5671809, https://doi.org/10.1155/2023/5671809.

In the article, there is an error in Figure 1d, where the BF, overNC panel was duplicated in the BF, shCYTOR panel. This error was introduced during the manuscript preparation by the authors. The correct Figure 1 is shown below:

We apologize for this error.

## Figures and Tables

**Figure 1:**
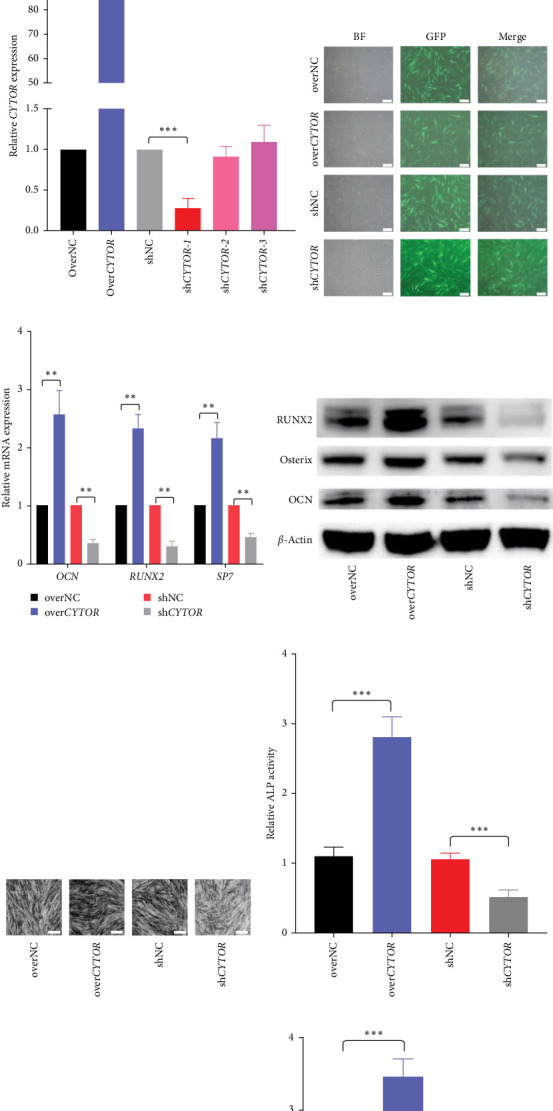
Long noncoding RNA CYTOR is mainly expressed in the cytoplasm of PDLSCs and facilitates the osteogenic differentiation of PDLSCs. (A) The expression and subcellular location of CYTOR in PDLSCs was observed by RNA fluorescence in situ hybridization. U6 was a reference gene which mainly expressed in the nuclei. Probe against U6 as a positive control. Cell nuclei were counterstained with DAPI (blue). Scale bar: 20 μm. (B) Relative level of CYTOR at indicated timepoints during osteogenic differentiation of PDLSCs were detected by qRT-PCR. (C) RNA level of CYTOR of PDLSCs treated with lentiviruses as indicated was measured by qRT-PCR. (D) The GFP signals were detected by inverted fluorescence microscope. (E) The mRNAs levels of osteogenic-related markers were determined by qRT-PCR. (F) The protein levels of osteogenic-related markers were measured by western blot. (G, H) The ALP activity was determined by ALP staining (G) and ALP measurement (H). (I) The mineralized nodules were assessed by Alizarin red staining. (J) Semiquantification of mineralized nodules. *∗p* < 0 : 05, *∗∗p* < 0 : 01, and *∗∗∗p* < 0 : 001, compared with the control group as indicated.

