# Supplementary material for: lncRNA CYTOR Facilitates Osteogenic Differentiation of Human Periodontal Ligament Stem Cells by Modulating SOX11 via Sponging miR-6512-3p
Source: Stem Cells Int. 2023 Mar 3;2023:5671809. doi: 10.1155/2023/5671809 (PMC10005871; doi:10.1155/2023/5671809)
Supplement: Supplementary Materials — Supplementary Figure 1: miR-6512-3p was downregulated in hPDLSCs after osteogenic induction and could be reduced by overexpression CYTOR. (A) Relative expression of eight potential miRNAs in hPDLSCs cultured in growth medium (GM) and osteogenic medium (OM) for 14 days were detected by qRT-PCR. (B) Levels of these eight miRNAs in hPDLSCs treated with indicated lentiviruses were detected by qRT-PCR. ∗∗p < 0.01, compared with the indicated control group. Supplementary Table 1: primers for quantitative real-time polymerase chain reaction. [file 5671809.f1.zip › Supplementart Figure 1 of ID5671809.docx]

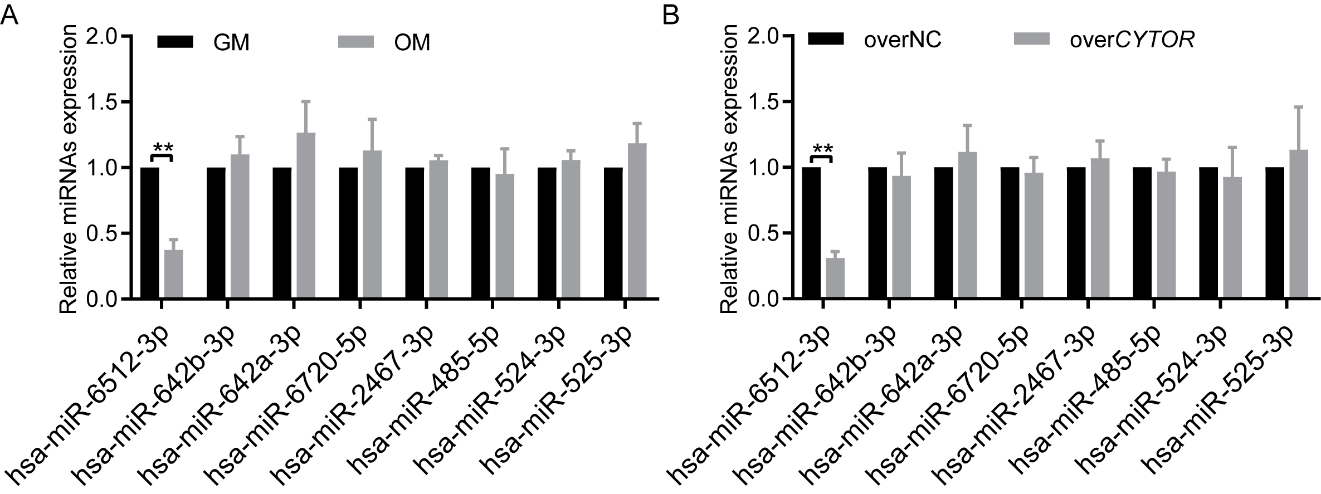


**Supplementary Figure 1. miR-6512-3p was down-regulated in hPDLSCs after osteogenic induction, and could be reduced by overexpression *CYTOR*.** (A) Relative expression of eight potential miRNAs in hPDLSCs cultured in growth medium (GM) and osteogenic medium (OM) for 14 days were detected by qRT-PCR. (B) Levels of these eight miRNAs in hPDLSCs treated with indicated lentiviruses were detected by qRT-PCR. ***p*<0.01, compared with the indicated control group.
